# Towards developing a model for the evaluation of hospital disaster resilience: a systematic review

**DOI:** 10.1186/s12913-020-4915-2

**Published:** 2020-01-29

**Authors:** Saeed Fallah-Aliabadi, Abbas Ostadtaghizadeh, Ali Ardalan, Farin Fatemi, Bijan Khazai, Mohammad Reza Mirjalili

**Affiliations:** 1 Department of Health in Emergencies and Disasters, School of Public Health, Tehran University of Medical Sciences, Tehran, Iran; 2 Harvard Humanitarian Initiative, Harvard University, Cambridge, USA; 3 Research Center for Health Services and Technologies, Semnan University of Medical Sciences, Semnan, Iran; 4 Karlsruhe Institute of Technology (KIT), Center for Disaster Management and Risk Reduction Technology, Karlsruhe, Germany; 5 Civil Engineering Department, Yazd University, Yazd, Iran

**Keywords:** Disaster, Hospital, Resilience, Structural and non-structural systems, Indicators

## Abstract

**Background:**

Hospitals play a vital role in disaster stricken regions. The resilient hospitals will be able to provide essential services to affected people and it can mitigate the risk of injuries during and after disasters. This study aimed to obtain the indicators required for the evaluation of hospital resilience.

**Methods:**

This systematic review was conducted in 2018. Through this systematic review, international electronic databases were investigated for the research studies published in English. The exclusion and inclusion criteria were determined to extract the hospital resilience indicators. These indicators will be used in order to develop a model to keep the system performance at an acceptable level during disasters.

**Results:**

Out of 1794 research studies published until September 2018, 89 articles and guidelines with full text were surveyed. Thirty-two articles and guidelines were then selected and analyzed to collect the indicators related to hospital disaster resilience (HDR). The domains and the indicators were extracted from these selected research studies. The authors collected and categorized them into three domains and twenty seven subdomains. The three domains included constructive, infrastructural, and administrative resilience. The relevant indicators were designed for each subdomain to assess HDR.

**Conclusion:**

Since diverse indicators affect hospital resilience, other studies should be conducted to propose some models or tools to quantify the hospital resilience in different countries and scopes with an all hazards approach.

## Background

Natural and human made disasters impose different range of damages, injuries and death to affected communities [1]. These can disrupt infrastructures and facilities, such as hospitals, schools, transportation systems, and emergency services. When these disasters occur, damages can be related to physical components, such as building structure, construction materials, and non-structural systems like medical equipment, lifelines, and architectural features. Hospital staff could be affected during disasters and their absence or unpreparedness influences the service continuity at urgent situations induced by disasters. Therefore, it is supposed that they would be well-aware of their role in implementing disaster plans. In case of hospital structural and non-structural failures, large investment should be done for continuity of service delivery in open areas or temporary buildings. Moreover, the activities for repair or reconstruction should be performed [2–4]. The literature review indicates that the main reason for most of the damages in health facilities is related to inappropriate site selection for the building, lack of proper design or insufficient maintenance [5]. Recent disasters throughout the world resulted in hospital damages and interruption in medical services [6–10]. For instance, in August 2012 and during Varzaghan-Ahar twin earthquakes in Iran, the performance of both Heriss and Ahar hospitals were not satisfactory. There were huge damages to the columns and beams, false ceilings and walls. Due to the lack of safety inside these two hospitals, the medical services were performed outside zone at the temporary hospitals set up in the tents [11]. After the devastating earthquake in Ezgele, Kermanshah in the west side of Iran in November 2017, the newly-built Islamabad and Sar-e-Pol-e Zahab hospitals were subjected to structural and non-structural damages which resulted in power outage and providing services outside the buildings [12]. The damages or malfunctioning of hospitals components in the case of emergencies and disasters will have direct or indirect impact on the continuity of medical services and result in more injuries or fatalities [13]. Therefore, it is important to encourage researchers, engineers and decision makers to develop ways to improve the resilience of healthcare facilities [14, 15].

In the world conference on disaster reduction in Hyogo, Japan, the aim of “hospitals safe from disaster” was proposed by ensuring that all new hospitals should be built with a level of resilience that strengthens their capacity to remain functional in disaster situations [16]. Also, during the third world conference held in Sendai-Japan, in 2015, the resilience of health infrastructures and disaster risk reduction measures were emphasized [17].

The systems resilience can be defined as containing four R that represents Robustness (inherent strength), Redundancy (replace ability of resources), Resourcefulness (having plans and strategies) and Rapidity (achieve priorities promptly) [18]. Ostadtaghizadeh et al., in a systematic review proposed a classification for community disaster resilience which included natural, economic, social, institutional, and infrastructural domains [19]. From the health aspect, the resilient system should be able to prepare for, withstand the stress of, and respond to the public health consequences of disasters successfully [20]. Hospital resilience is related to decreasing vulnerability to the shocks brought by disasters and increasing adaptive capacity brought by improved measures and opportunities [21].

There are significant amount of research conducted to understand hospitals response to hazards and some studies containing a tool or instrument like Hospital Safety Index (HSI) as a rapid, reliable, and cost-effective diagnostic tool for assessing the safety of hospital buildings, critical systems and equipment, the availability of supplies, and the emergency and disaster management capacities of the hospital. This tool not only helps to assess safety status, but also helps to evaluate the response capacity of the hospitals [22–27]. Moreover, there are some studies that mentioned instruments for assessing Hospital Disaster Resilience (HDR). In some of them the authors focused on operational characteristics of hospitals; however, the structural and non-structural aspects of hospital resilience were not mentioned in details [28–33]. For instance, Zhong proposed multiple concepts for assessing hospitals resilience in response to disasters in China. The variables used in this study included hospital safety, emergency services, surge capacity, command, disaster plan, logistics, staff ability, disaster training, communication and cooperation systems, recovery, and adaptation [34]. With regard to the importance of evaluating, monitoring, and planning in order to improve HDR, it is necessary to develop a validated model to evaluate HDR. To do so, it is required to identify all the factors and indicators mentioned in different studies and categorize them in a framework [33]. This study aimed to identify, collect, and categorize the factors that could be used for assessing HDR.

## Methods

### Databases and search strategy

This study as a systematic review covered the electronic academic resources, such as articles, books, documents, and reports published before September 1, 2018. There were no limitations with the type or the date of studies, but the study language was only restricted to English. International electronic databases, including Pubmed, Web of Science, Scopus, and Google scholar were investigated. In addition, the ProQuest Research Library which only contains thesis was searched too. The search key terms were selected from three major scopes after consulting with experts, hospital, disasters, and resilience. In addition to keywords provided by experts, to find more relevant citations, MeSH entry terms service of PubMed were used. The experts mainly were from scientific institutions and organizations which were involved in disaster risk reduction and disaster management and had some academic papers and also systematic review articles in this field. The search strategy was determined for searching the databases as follows:

(Disaster* OR emergency*) AND (resilience*) AND (hospital* OR healthcare OR health care). This strategy was applied in titles, abstracts, keywords in all databases. The complete search strings are included in Additional file 1.

### Inclusion and exclusion criteria

Included documents were credible articles, guidelines, and grey literature written in English that focused on structural or non-structural systems of a hospital, such as buildings, lifeline or utility, including water, power, and fuel/gas/energy. Moreover, they had to explain or present the factors, indicators, variables, models or instruments that affected the resilience of the structural or non-structural systems of the hospital in case of disasters. The articles which were related to the individual, staff, psychological and economic resilience were excluded and also those which did not present the factors or indicators of HDR. The documents without full text or those which their full texts were not available were excluded. Table 1 shows the inclusion and exclusion criteria.

**Table 1:** The inclusion and exclusion criteria for article selection

Inclusion Criteria	Articles, guidelines and grey literature written in English.
	The studies that focused on structural or non-structural systems of a hospital.
	The studies that present the factors, indicators, variables, models or instruments that affected the resilience of the structural or non-structural systems of the hospital.
Exclusion Criteria	The studies that relates to fields of resilience such as individual, staff, psychological and economic resilience.
	The articles that do not present the factors or indicators of HDR.
	The studies that we couldn’t find their full text.

**Fig. 1:**
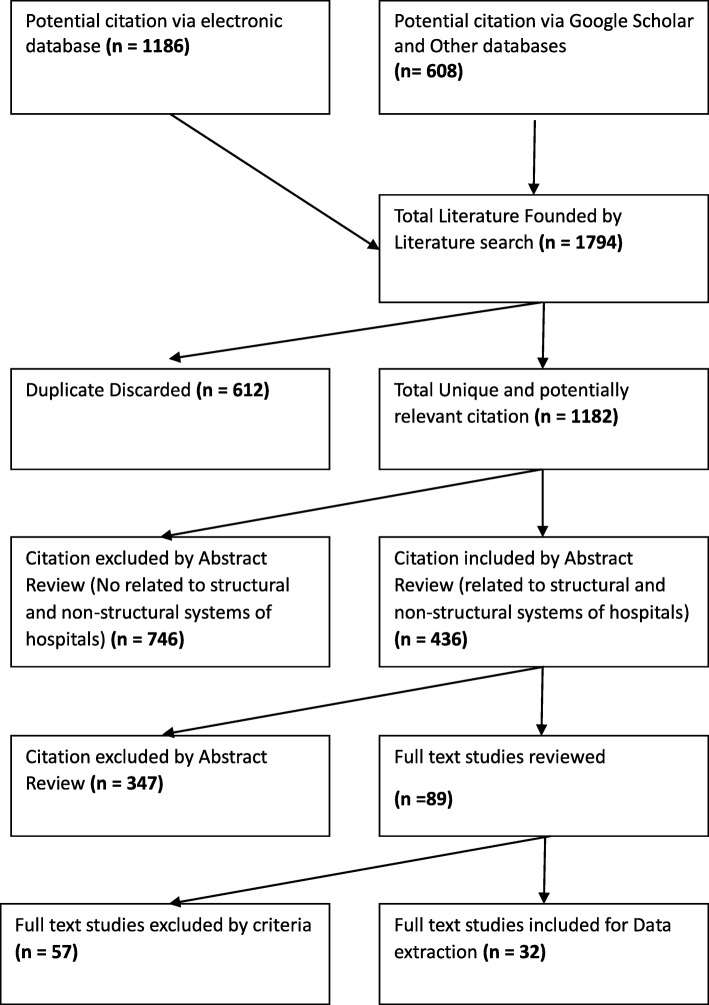
Flow diagram of the search and selection of studies

Figure 1 outlines the PRISMA flow diagram for the selection process in the studies for this review.

### Data extraction and analysis

For extracting the data, two independent researchers performed the screening of the titles and abstracts to choose the relevant articles according to the inclusion and exclusion criteria. Then, the full texts of the total articles were reviewed. In the case of disagreements between two researchers at this stage, the third researcher joined the review team and helped them select the most relevant articles. Then, two forms were developed, one for importing general information from the selected articles by mentioning the names of the authors, type of articles, the research country, date of publication, methodology, and objective of the studies. Another form was applied for identifying the name of the models and tools, the details of domains and indicators and the factors mentioned in them.

## Results

The initial search resulted in identification of 1794 potentially relevant documents from the four international databases (PubMed, Scopus, Web of Sciences and Google scholar), 612 literatures were duplicated and removed. The remained documents included 1182 studies, from which 746 were excluded after screening their titles and abstracts; since they did not include the determined inclusion criteria; while 436 papers were included. By investigating the abstracts in details, 347 studies were excluded because of having no domains or indicators of HDR. Then, 89 studies were selected for full text reading and 57 articles were excluded due to lack of enough information or suitable factors or indicators to assess HDR or the full texts were not accessible. Finally, full-text review of these articles led to 32 documents which were included in the present study. The study was developed based on the PRISMA checklist assessment tool.

### Descriptive analysis

By reviewing the 32selected articles and guidelines published before September 1, 2018, it was obvious that the largest numbers of papers were from the United States (40%), followed by the United Kingdom and Malaysia (10%), Iran, China and Canada (7%).Other research studies were from Colombia, Belgium, Italy, Japan, Switzerland, Australia, and New Zealand. These 32 final documents included original articles, guidelines, reviewed articles, and conference papers. It also showed that the focus on HDR has recently increased, so that more than 20 included articles in this systematic review have been published after 2014. Sixteen articles had the all-hazards approach and others (10 articles) discussed the seismic resilience of hospitals and six articles were related to climate change, including extreme weather events, hurricane or flood. Through surveying, the methodology of the articles and guidelines showed that only two articles were review research studies, four guidelines, and the remained articles were original research studies. Literature review was as a basic part of all of the articles. The methodology of these articles were divided into three types, including eight articles which used qualitative methods, 13articles used quantitative methods, and the methodology of the other nine papers were both qualitative and quantitative. The remained article was a narrative study. Additional file 2 shows the characteristics of the full sources included in the study [4, 8, 25, 27, 29, 31–33, 35–58].

### Thematic analysis

For extracting the domains, subdomains, and indicators related to HDR, all the 32 selected articles and guidelines were reviewed. From these studies, four research studies proposed a model, framework or tool which developed the process of assessing HDR. These model, tools, and framework were as follows: Hospital Safety Index (HSI) [27], Dynamic approach to the seismic resilience of hospitals [45], Measuring framework of the hospital resilience [33], and The indicators for assessing hospital disaster preparedness in Japan [25]. Some of the articles did notpresent any tool or model for assessing HDR; however,they consisted relevant indicators or variables to HDR. Table 2 represents tools and models, as well as indicators for assessing HDR.

**Table 2:** Domains and indicators related to hospital resilience in the studied articles

Tool 1: Hospital Safety Index (HSI) [27]	
Domains	**Indicators**
Structural	Structural system design, Construction materials, Interaction of nonstructural elements with the structure, Proximity of buildings, Structural redundancy, Structural integrity, Irregularities in the building, Safety, and the condition of connections, column, beam and, foundations.
Non- Structural	Condition and safety of elements, Layout of hospital’s critical services and equipment, Safety of access routes, Emergency exit and, evacuation routes, Condition and the safety of the utility services, equipment, furniture, and lighting systems, Emergency maintenance and restoration of the utility systems, Water and power supplies for hospital services, Fire/smoke detection systems, Fire suppression systems, Water supply for the fire suppression, Safety of the hazardous materials, Fuel reserves.
Functional	Coordination of the emergency and disaster management activities, Hospital emergency and disaster plans, Communication and information management, Human resources, Logistics and finance, Patient care and support services, Evacuation, Decontamination and security.
Tool 2: Indicators for Assessing Hospital Disaster Preparedness in Japan(25)	
Domains	**Indicators**
Structural	Earthquake and fireproof building construction, Suitable layout of hospital building according to background natural hazards of region, Available space for emergency evacuation.
Non- Structural	Medicine/chemicals/potential hazardous substance managements, Material safety data sheets (MSDS).
Functional	Medical equipment for emergency medical services, In-house power generator, Drinking water, Food, Folded beds, Communication tools, Helipad space, Accessibility (roads), Vehicles for disaster medical assistance team.
Human Resources	Availability of personnel, Disaster education, Professional training for the emergency medical service, disaster exercise for hospital staff/workers and patients.
Model 1: System Dynamics Approach to the Seismic Resilience Enhancement of Hospital(45)	
Domains	**Indicators**
Endogenous	Patients in backlog, Functional capacity, Patients in hospital, Average treatment time, Total required monetary resources, Monetary resource allocation rate, Building damage, Number of active staff, Medicine inventory.
Exogenous	Earthquake intensity, Reserved monetary resources, Patient arrival rate, Initial condition, Effects of deficiency on average treatment time/Fatality rate.
Excluded	Transportation service ability, Condition of supporting Lifelines, Performance of the other hospitals in the region.
Framework1: Framework for Measuring the Hospital Disaster Resilience (33)	
Domains	**Indicators**
Vulnerability and safety	Disease surveillance, Hospital infrastructural safety and vulnerability.
Disaster preparedness and resources	Emergency leadership, Community cooperation and communication, Disaster plans, Disaster stockpiles and logistics management, Emergency staff, Emergency training and, drills.
Continuity of essential service	Emergency medicine, Surge capacity.
Recovery and adaptation	Recovery capability, Evaluation and adaptation.
Other indicators	
Integration of utilities in resilience codes and legislations [37], Hospital retrofitting, Laboratory test, Withstand disaster-induced damage and disruption, Seismic design level, Cost of reconstruction, [39], Desired performance level, Occupancy or usage type, Direct and indirect economic loss [41, 42], Societal preparedness, public policies [42], Dependency of Lifeline, Repair resources [43], Personnel participation in emergency planning, List of personnel contact information, Management and mobilization of volunteers, Agreement with suppliers [45], Continuous electric load analysis and monitoring, Redundancy for most critical areas, Automatic test facilities [51], Updated hospital building documents/ drawings / plans, Internal circulation and interoperability, Hospital standard operating procedures (SOP), Hospital emergency operations center (EOC), Establishing the incident command system (ICS) [53], Providing water from outside sources, prioritizing water consuming activities, Providing contingency plans [54], Permit and clearance [55], Protecting from cyber-attack, Monitoring systems for utility system failure [35], Participation of donors community, Promote research and studies [36], Amount of consumable commodity [58], Cash to purchase supplies and services, Interoperable communications, Management and maintenance of community- based outpatient clinic emergency operations plan, Specialty outpatient services, Provision of ambulatory clinical services, employees welfare, reporting incident system [27], Time under care [40], Soil Type, Ductility, Strength and Stiffness, Configuration: Size and Shape, Structural Systems selection, Interstitial space for utility installations, Design flood elevation, Flood hazard maps [34], Hospital risk assessment, Thermal mass, Insulation, Reduced stress and fatigue [46], Accreditation standards, Community preparedness, Mitigation budget, Onsite technical expertise, Mutual aid agreements, Homecare infrastructure, Ability to discharge noncritical patients, Facility age, Length of power outage, Statue of other infrastructures, Time of power failure, Statue of organizational robustness, Statue of technical robustness [47].	

## Discussion

This study aimed to determine the domains and indicators for assessing HDR through a systematic review. Given that the proper and timely operation of hospitals is crucial in times of crisis; their resilience needs to be addressed. Therefore, the comprehensive assessment of HDR helps to find the weaknesses and challenges in the scope of disaster risk and remove them to mitigate the harmful consequences of disasters [59]. The desirable performance of hospitals during and after disasters and their continuity to services depends on different factors, such as hospital building stability, including the structural and non-structural systems [60]. Several studies were carried out in different fields of HDR, such as organizational resilience which is related to functional services of the hospital [1, 46, 61–63]. However, the studies which are substantially related to structural and non-structural components of hospitals have been rarely found. Thus, a systematic method of HDR regarding the structural and non-structural systems would be required. Some indicators which were directly related to medical services, such as triage or referral, transfer, and reception of patients excluded in the present review. However, the indicators that described the structural and non-structural characteristics as well as administrative and functional activities concerning structural and non-structural systems included in this study. Most of the studies focused more on electrical and water utilities and transportation networks in hospitals [40, 42, 46, 51, 53, 54, 56, 58, 64]. The literature showed that other utilities in hospitals like communication system, gas supply system, sewage system as well as non-structural components of buildings, such as architectural elements have been less considered. In the case of healthcare facilities, nonstructural components often represented greater economic value rather than the structure itself. Analyses indicated that nonstructural components generally accounted for more than 80% of the total costs of a hospital [65]. Moreover, there is a crucial difference between risk reduction (safety and preparedness) and resilience in this regard. Safety is defined as “a state in which hazards and conditions leading to physical, psychological or material harm are controlled in order to preserve the health and well-being of individuals and the community” [66]. However, resilience is defined as a concept for the ability or capacity of a system or community to deal with risk [67]. Based on these definitions, it can be found that safety and risk reduction is mainly used to reduce the level of risk; however, resilience is used to keep control of the functionality of a system when the system is prone to risk. The model, tools, and framework in Table 2 had properties which helped to improve a model to assess HDR. The HSI tool and frameworks proposed by Zhong had an all-hazards approach [27, 33]. The HSI has three sections, including structural, non-structural and disaster management system [27]. The structural system refers to elements of building that withstand loads. Other elements of buildings, such as utilities and architectural systems are categorized as non-structural system. One of the advantages of this tool is that the non-structural section is wide and consists of many sub-categories. The disaster management category in HSI has emphasized on preparedness of the hospital system, including human resources readiness, preparing action plans, management of communication and information systems, patient care and support services, and logistics and finance. Another surveyed tool was presented by Mulyasari et al., including four domains and indicators for improving the resilience of hospitals against earthquakes in Japan [25]. Analyzing this tool demonstrates that the proposed approach is not comprehensive in spite of having four domains. Three domains similar to HSI modeland human resources were also added. This tool focuses mainly on the power and water systems and not structural condition of hospitals and other utilities. Moreover, the main focus of human resources domain is just on medical staff, so that the other groups of hospital staff have been neglected. The other disadvantage is that this tool considers only the preparedness phase, while a resilient hospital system should cover different phases of disaster management, including mitigation, preparedness, response, and recovery [68]. The model proposed by Khanmohammadi et al., concentrates on the hospital building and relevant technical services failure after earthquakes at the recovery phase in Iran. It cites the impacts of hospital damages and the resource shortage on the quality of services and uses the relevant variables to quantify the hospitals resilience [45]. The model variables were classified into three groups, including the endogenous, exogenous, and excluded variables. The endogenous variables can affect the building damages, the exogenous variables, including earthquake intensity, and the excluded variables that would help to quantify the functionality of the hospital.

Zhong suggested a framework including four domains and 12 subdomains for assessing hospital resilience assessment in China [32–34]. This framework highlights managerial aspect of hospitals more than the structural and non-structural systems at the time of danger. Continuity of essential medical services as one of the domains of this framework only takes two factors, i.e. emergency medicine and surge capacity; whereas service continuity should include utility services, staff participation, and other similar fields as well [34]. In this framework, all the building elements have been mentioned as the architectural components and there is no distinction between the windows and doors with medical and laboratory equipment or electrical installations. Moreover, the financial supports of the hospital system has been neglected in the mentioned framework [33].

The idea of this study is to extract the relevant indicators which would be able to measure them quantitatively in the developed model for removing the weak points of qualitative models.

By considering all advantages and disadvantages of HDR surveyed models and tools, the indicators extracted from research studies in this systematic review were collected and categorized in Table 3. These indicators can be useful for assessing HDR.

**Table 3:** Domains, sub-domains and indicators of hospital disaster resilience

Domain	Sub-Domain	Indicators
Constructive Resilience	**Stability**	Building Structural System, Retrofitting the building, Construction materials, Structural redundancy, Laboratory test results, Plan and vertical irregularities, Structural configuration and lateral resistance system, Structural integrity of the building, Building’s age, Soil type, Reduced stress and fatigue, Withstand disaster-induced damage and disruption
	**Design**	Open spaces, Hazards maps and zones, The space between buildings, Proper zoning of building areas, Building regulations and design codes, Hospital design and layout (location, slope, sea level, water ground, seismicity, configuration etc.), Permit and clearance process, Interstitial space for utility installations, Occupancy or usage type of different parts of hospital
	**Architectural**	Safety of internal path (stairs, corridors and, elevators), Safety of the architectural elements such as doors, windows, internal and exterior walls, facings etc.
	**Transportation and Transition System**	Space for the ambulance stopping and passing, Safety of the access routes, Space for the helicopter landing, Capacity of hospital parking, Ramps for moving patients’ bed and for the people with disabilities.
Infrastructural Resilience	**Power**	Maintenance and safety of the electrical power systems, lightning systems and the generators, Automatic test equipments, Power conservation activities, The age of power systems, Continuous electric load analysis and monitoring, Redundancy for most critical areas, Monitoring systems for the power outage or power failure, Protecting from terrorist attack, Length of power outage.
	**Water and Sewage system**	Maintenance and safety of the water system and the sewage system, Water saving and conservation activities, Time and length of water interruptions, The age of water and sewage system, Providing water from outside sources, Automatic test equipments, Plans for prioritize critical water consuming activities, Monitoring systems for water outage or water contamination,
	**Communication and IT System**	Maintenance and safety of IT and Communicationsystem, Automatic test equipments, Maintenance of alternative and backup communication and, IT system.
	**Heating, Ventilation and air conditioning (HVAC)**	Maintenance and safety of HVAC systems, Automatic test equipment, Monitoring systems to provide warning of HVAC system failure.
	**Fuel**	Fuel reservation, Safe location of the fuel storage.
	**Medical gas**	Maintenance and safety of the medical gas system, Providing alternative sources of medical gases.
	**Equipment and furniture elements**	Equipment anchorage and fixing, Safety of rooms’ furniture and equipment, Safety of medical and laboratories equipments.
	**Hazardous Material**	Hazardous material forms and documents, Safety of hazardous solid waste, wastewater, and liquid waste.
	**Fire system**	Condition and safety of the fire systems, Water supply for fire protection.
Administrative Resilience	**Disaster Plan**	Emergency preparedness, Emergency response, Contingency plans, Emergency regulations, Emergency recovery, Emergency evacuation, Standard operating procedures (SOP).
	**Risk assessment and reduction**	Hazards identification and analyses, Hospital initial condition, Structural and non-structural risk reduction measures, Estimation of structural and non-structural damages, Estimation of losses due to an interruption in services, Developing the measures for risk reduction in future, Amount of consumable commodity.
	**Response**	The proper response, Timely response, Early Warning, Ability to discharge noncritical patients.
	**Command**	Establishing the incident command system (ICS).
	**Coordination**	Create a framework for the participation of local authorities, Effective implementing information and communication system, Access of operational personnel to expert opinions, Personnel partnership in finding solutions for defections, The coordination between different parts of the hospital. Accreditation standards, Performance of the other hospitals in the region. Dissemination of Personnel Incident Information to Staff During an Incident.
	**Evacuation**	Proper and timely response according to the emergency evacuation plans.
	**Need assessment**	Medical services demands, The rate of patient arrivals.
	**Logistic and supplies**	Logistic management of requirements, Personnel recalling and transmitting system, Agreement with suppliers, Resources availability, Mental health and psycho-social treatment for patients, families, and health workers, Details of the types, amounts and quality of the equipment and stocks, Applying alternative systems in a safe mode.
	**Safety committee**	Establishing and developing the emergency committee, List of personnel contact information, Human protection from fire, chemical and radiological hazards, Infection Surveillance, Prevention and control procedures, Insurance status.
	**Continuity of services**	Preventing reduction in the system operation, Ability to adapt timely to emergency state, Self-organization and re-structuring.
	**Volunteers**	Consider community-based activities, management and mobilization, Societal preparedness, Participation of donors community
	**Finance**	Management of the disaster financial and administration system, Direct and indirect economic loss, Cost of repair and reconstruction, Mitigation budget.
	**Recovery**	Maintenance and repair plan for equipment, Priorities of repair and reconstruction, Psychosocial services for emergency staff.
	**Training**	Educational courses, Exercise, Promote research and studies, Lesson learned from past disasters.

Totally, the collected indicators were categorized in 3 domains, 27 subdomains, and relevant indicators that can be used for assessing HDR in future studies. The domains in Table 3were divided into three resilience types, including constructive, infrastructural, and administrative. Constructive resilience as a domain encompasses all elements of hospital building. This domain consists of architectural elements and the design of spaces and structures as subdomains for optimum function of hospitals to be inherently flexible, strong, and adaptive to emergency situation. Another subdomain is transportation and transmission that should be designed before the hospital construction and facilitates the access of patients and staff to the hospital. The infrastructural resilience consisted of non-structural elements which facilitate the hospital functions. The utilities and services, such as power, water or fire control were mentioned with their relevant indicators in this section. In addition, the protection of electrical utilities from terrorist and cyber-attacks was highlighted as a subdomain in the infrastructural resilience. The administrative resilience domain included activities for disaster management hospital, such as hazard and vulnerability reduction measures, preparedness, response, and recovery plans. In this domain, managing the volunteers is also a critical subdomain which shows the importance of the community-based activities as well as participatory approach of resilience. Due to the importance of repair and reconstruction of the structural and utility systems, the cost and priorities of these actions were mentioned as finance and recovery in the administrative resilience domain. Also, the domains and subdomains have the potential to substitute according to 4R, including resourcefulness, redundancy, robustness, and rapidity. For instance, infrastructural resilience as a domain was categorized into the resourcefulness and redundancy as the resilience criteria demonstrate the hospital capability for mobilizing alternative external resources. It can also involve human resources and material in the process of recovery and also to substitute alternative elements. Constructive resilience is associated with robustness as another resilience criterion which shows the ability of hospital system to withstand a given level of shocks. Extracted indicators relevant to recovery and response are accounted as rapidity which is one of the resilience criteria reflecting the capacity of hospital system to meet priorities in order to recover functionality and avoid future disruption [39, 42, 64].

### Limitations

The main limitation of this review lies in the fact that English articlesand documents were only included. Therefore, the authorsmay have lost some of the relevant research studies which were in other languages. Furthermore, there were limited access to the full text of some papers that could affect finding comprehensive indicators. Identification and extracting indicators in some articles, especially in engineering fields, was difficult. Moreover, the number of extracted indicators wereconsiderably high that the authorshad to merge similar indicators.

## Conclusions

This study highlighted the role of indicators and defined domains in order to assess hospital resilience through an integrated model. To do so, a set of domains, subdomains, and relevant indicators were extracted to be able to measure HDR quantitatively in future studies. The literature review proves that the functional safety has been an interested topic among scholars in order to increase the hospital resilience. However, hospital building and spaces as constructive resilience, also lifelines as infrastructural resilience,and importantly services as administrative resilience playsignificant role in hospital performance during disasters. Thesedomains and subdomains have beenignored in some studies. However, this studyrelies on the three mentioned resilience domains to focus onhospital resillience. Moreover, measuring HDR quantitatively is one of required factors suggested to be achieved in other studies as an important issue.

Further studies should be done to select other related indicators usingexpert judgement and improvement of the existing models. In addition, the validity of the model and indicators should be verified in further studies due togeneralizability in different countries and diverse hazards. These tools or models can help societies and government officials to reduce hospitals vulnerability and improve their performance and resilience against disasters.

## Supplementary information

**Additional file 1.** Database Search Strategy.

**Additional file 2. **Characteristics of the articles and other sources included in a systematic review of the literature**.**

## Data Availability

The data supporting the conclusions in this article are available in the additional files. Data supporting study findings are available upon request.

## Abbreviations

HDR: Hospital Disaster Resilience
HSI: Hospital Safety Index
PRISMA: Preferred Reporting Items for Systematic Reviews and Meta-Analyses
