# Supplementary material for: Towards developing a model for the evaluation of hospital disaster resilience: a systematic review
Source: BMC Health Serv Res. 2020 Jan 29;20:64. doi: 10.1186/s12913-020-4915-2 (PMC6988294; doi:10.1186/s12913-020-4915-2)
Supplement: Supplementary file 1 — Additional file 1. Database Search Strategy. [file 12913_2020_4915_MOESM1_ESM.docx]

**Additional file 1:** Database Search Strategy.

### All searches before September 1, 2018

### PUBMED

### (disaster*[tiab] OR emergenc*[tiab] OR catastrophe*[tiab] OR earthquak*[tiab] OR flood*[tiab] OR hurricane*[tiab] OR tsunam*[tiab] OR typhoon*[tiab] OR landslide*[tiab] OR avalanch*[tiab] OR storm*[tiab] OR tornado*[tiab]) AND (resilien*[tiab] or resilient or resiliency) AND (hospital[tiab] OR healthcar*[tiab] OR "health care"[tiab] OR (health[tiab] AND care[tiab]) OR (Tertiary[tiab] AND Care[tiab]))

### WOS

### TS=(disaster* OR emergenc* OR catastrophe* OR earthquak* OR flood* OR hurricane* OR tsunam* OR typhoon* OR landslide* OR avalanch* OR storm* OR tornado*) AND TS=(resilienc* OR resilient or resiliency) AND TS=(hospital* OR healthcar* OR "health care*" OR (health AND care*) OR (Tertiary AND Care*))

### SCOPUS

### (TITLE-ABS-KEY(disaster*) OR TITLE-ABS-KEY(emergenc*) OR TITLE-ABS-KEY(catastrophe*) OR TITLE-ABS-KEY(earthquak*) OR TITLE-ABS-KEY(flood*) OR TITLE-ABS-KEY(hurricane*) OR TITLE-ABS-KEY(tsunam*) OR TITLE-ABS-KEY(typhoon*) OR TITLE-ABS-KEY(landslide*) OR TITLE-ABS-KEY(avalanch*) OR TITLE-ABS-KEY(storm*) OR TITLE-ABS-KEY(tornado*)) AND (TITLE-ABS-KEY(resilien*) OR TITLE-ABS-KEY(resilient or resiliency)) AND (TITLE-ABS-KEY(hospital*) OR TITLE-ABS-KEY(healthcar*) OR TITLE-ABS-KEY("health care*") OR (TITLE-ABS-KEY(health) AND TITLE-ABS-KEY(care*)) OR (TITLE-ABS-KEY(Tertiary) AND TITLE-ABS-KEY(Care*)))
