# Supplementary material for: Towards developing a model for the evaluation of hospital disaster resilience: a systematic review
Source: BMC Health Serv Res. 2020 Jan 29;20:64. doi: 10.1186/s12913-020-4915-2 (PMC6988294; doi:10.1186/s12913-020-4915-2)
Supplement: Supplementary file 2 — Additional file 2. Characteristics of the articles and other sources included in a systematic review of the literature. [file 12913_2020_4915_MOESM2_ESM.doc]

**Additional file 2**: Characteristics of the articles and other sources included in a systematic review of the literature

| Method | Study Objective | Disaster Type | Type | Year | Country | 1st Author | No. |
| --- | --- | --- | --- | --- | --- | --- | --- |
| Qualitative and Quantitative | - Exploring the challenges facing healthcare facilities function especially those related to the utility supplies. - Development of a model to quantify the impact of the utility supplies interruption on the operation of healthcare facilities. | Earthquake | Original research | 2014 | UK | Achour (38) | 1 |
| Qualitative | Vulnerability assessments and risk analysis for protecting the health sector infrastructure. | All Hazards | Original research | 2008 | USA | [Barror](https://www.researchgate.net/scientific-contributions/2095573722_RF_Barror?_sg=IuJkz3XYiTbVZD0GM1YgCj1PakwjByt-R8jU_dWnS3MwVUKj5OpcKatX5F9ZUFG7SYz7s4w.Jol7meXERhCR2ljgVC3_K9b2wxhzdXKvJ6sqlWzzunlPsEdp0ZXcjU_vhoDfrEzCdtTpVIcekxrCKaaz0iFF0g)(39) | 2 |
| Quantitative | Quantify the seismic resilience of the acute care facilities. | Earthquake | Original research | 2007 | USA | Bruneau(40) | 3 |
| Qualitative | Survey the impacts of the extreme weather events on the management of healthcare facilities. | Extreme weather | Original research | 2012 | Australia | Chow(41) | 4 |
| Quantitative | Quantify disaster resilience of systems. | Earthquake | Original research | 2010 | USA | Cimellaro(42) | 5 |
| Quantitative | Survey the influence of the damages of structural and non-structural components on the hospital organizational systems. | Earthquake | Original research | 2010 | USA | Cimellaro(43) | 6 |
| Qualitative and Quantitative | Developing a preparedness assessment tool for hospitals. | All Hazards | Original research | 2016 | USA | Dobalian(29) | 7 |
| Qualitative | Help the hospital administrators and facility managers in implementing mitigation measures to decrease the vulnerability of the hospi­tals to natural disasters. | Earthquake, Floods, and High Winds | Guideline | 2007 | USA | Federal Emergency Management Agency (FEMA)(35) | 8 |
| Quantitative | • Study community resilience and recovery after major seismic events.  • Investigate the factors causing the reduction of the hospital services after the seismic hazard. | Earthquake | Original research | 2017 | USA | Hassan (44) | 9 |
| Qualitative | Guidance for planning for a resilient healthcare. | All Hazards | Guideline | 2014 | UK | HBN(36) | 10 |
| Qualitative and Quantitative | Assess the loss of the function of facilities and the disaster impacts to the structure, staff and supplies. | Earthquake | Original research | 2014 | New Zealand | Jacques(8) | 11 |
| Quantitative | Identify the recovery plan that develops hospital resilience against future earthquakes. | Earthquake | Original research | 2018 | IRI | Khanmohammadi(45) | 12 |
| Qualitative | Assess the hospitals’ disaster preparedness along with the key measures of the hospital resilience. | Typhoon | Original research | 2017 | USA | Labarda(4) | 13 |
| Qualitative | Recommend tools and processes to improve resilience for the nursing homes. | All Hazards | Original research | 2017 | USA | Lane(46) | 14 |
| Qualitative and Quantitative | Define resilience for the hospitals and propose resilience indicators for assessing the current situation of the hospital resilience. | Climate change | Original research | 2011 | UK | Masko(47) | 15 |
| Qualitative | Find applied decisions that develop infrastructure resilience to extreme events. | All Hazards | Original research | 2008 | Canada | McDaniels(48) | 16 |
| Qualitative and Quantitative | Evaluate the earthquake preparedness of hospitals in eight Japanese cities. | Earthquake | Original research | 2013 | JPN | Mulyasari(25) | 17 |
| Qualitative | Identify hospitals' capacity to respond effectively during a disaster or crisis. | All Hazards | Original research | 2018 | Malaysia | Norazam(49) | 18 |
| Qualitative | - Assess hospitals’ safety and vulnerabilities. - Make recommendations on necessary actions. - Promote measures for improving the safety and developing emergency preparedness. | All Hazards | Guideline | 2015 | Switzerland. | Pan American Health Organization  (PAHO)(27) | 19 |
| Qualitative and Quantitative | Develop the checklist and recommendations for operationalization of the toolkit in Canada and internationally. | Climate change | Original research | 2014 | Canada | Paterson(50) | 20 |
| Qualitative | - Surveying the hospital's reaction to power loss following a disaster. - Surveying the ways that the typical power system’s layout is resilient. | All Hazards | Original research | 2017 | Italy | Prudenzi(51) | 21 |
| Narrative | Assess and develop the existing tools for measuring the hospital resilience. | All Hazards | Systematic review | 2015 | Belgium | Rodriguez-Llanes(52) | 22 |
| Quantitative | Establish a relationship between the hospital resilience indicators and the preparedness attributes. | All Hazards | Original research | 2017 | Malaysia | Samsuddin(53) | 23 |
| Qualitative and Quantitative | Present a new framework for evaluating the disaster management based on the resilience principles in the hospital systems in a developing country. | All Hazards | Original research | 2015 | IRI | Shirali(31) | 24 |
| Qualitative | Explain the methods of planning to prevent internal hospital disaster. | All Hazards | Special Report | 2004 | USA | Sternberg(54) | 25 |
| Qualitative and Quantitative | Evaluate the content validity of the disaster resilience hospital assessment tool. | All Hazards | Original research | 2016 | Malaysia | Takim(55) | 26 |
| Qualitative | Develop a checklist of actions for healthcare and public health centers to strengthen the resilience of their community’s health sector to disasters. | Hurricane | Original research | 2017 | USA | Toner(56) | 27 |
| Quantitative | Evaluate the seismic risk of facilities related to the vulnerability of the hospital structural and non-structural components with the response to their capacity. | All Hazards | Original research | 2014 | Colombia | Valcárcel(57) | 28 |
| Quantitative | Planning guidance for decision-makers about the ways to make hospitals more resilient against the possible disruption scenarios. | All Hazards | Original research | 2014 | USA | Vugrin(58) | 29 |
| Qualitative | Protect the lives of patients and health workers by ensuring the structural resilience of health facilities.  Make sure health facilities and health services continue to service after disasters. | All Hazards | Guideline | 2007 | UN | World Health Organization (WHO)(37) | 30 |
| Qualitative | Define a conceptual framework and approaches to measure hospital resilience. | All Hazards | Review | 2014 | CHN | Zhong(33) | 31 |
| Qualitative and Quantitative | Develop a framework of key indicators of hospital resilience. | All Hazards | Original research | 2014 | CHN | Zhong(34) | 32 |
